# Delayed aortic regurgitation after TEVAR procedure: a case report

**DOI:** 10.1186/s13019-022-02083-3

**Published:** 2022-12-23

**Authors:** Soo Jin Park, Seungwook Lee, Jeong A Son, Seungji Hyun, Do Jung Kim, Sang Hyun Lim, You Sun Hong

**Affiliations:** 1 Department of Thoracic and Cardiovascular Surgery, Asan Medical Center, 88, Olympic-ro 43-gil, Songpa-gu, Seoul, 05505 Korea; 2 Department of Thoracic and Cardiovascular Surgery, Ajou University School of Medicine, 164, Worldcup-ro, Yeongtong-gu, Suwon, 16499 Korea

**Keywords:** Aortic regurgitation, Catheter induced injury, Thoracic endovascular aortic repair (TEVAR)

## Abstract

**Background:**

Acute aortic regurgitation (AR) is uncommon condition and usually results in an emergent situation because the left ventricle does not adapt quickly due to a sudden increase in end-diastolic volume caused by the regurgitant flow. Thoracic endovascular aortic repair (TEVAR) is a procedure that places a stent-graft on the lesion of thoracic aorta through a minimally invasive approach.

**Case presentation:**

Here we report that a catheter-induced aortic valve injury associated with TEVAR can cause delayed AR, exemplified by the case of a patient who developed acute AR 42 months after TEVAR. For this, aortic valve replacement was performed and the patient was discharged without complications.

**Conclusion:**

Our results demonstrate that when a catheter-related procedure is performed around the aortic valve, slight injury of the valve can cause aortic insufficiency even 3 years after surgery. Consequently, when performing a catheter-related procedure around the aortic valve, special attention is always required.

**Supplementary Information:**

The online version contains supplementary material available at 10.1186/s13019-022-02083-3.

## Background

Aortic regurgitation (AR) is the diastolic regurgitation of blood from the aorta into the left ventricle (LV) through a dysfunctional aortic valve [1]. Acute AR is uncommon and may result from type A aortic dissection, endocarditis or trauma, and less commonly iatrogenic causes [2]. Acute severe aortic regurgitation usually results in an emergent situation because the left ventricle does not adapt quickly due to a sudden increase in end-diastolic volume caused by regurgitant flow [2]. If not surgically corrected, cardiogenic shock may be induced.

Thoracic endovascular aortic repair (TEVAR) is a procedure that places a stent-graft on the lesion through a minimally invasive approach in various pathology situations of thoracic aorta and thoracoabdominal aorta [3]. TEVER is recommended for saccular aneurysm that occurs in the isthmus portion of the aorta after trauma [4].

We would like to report that catheter-induced aortic valve injury used during TEVAR can cause delayed aortic regurgitation through a case of a patient who had acute aortic regurgitation 42 months after TEVAR and was discharged after performing surgical aortic valve replacement.

## Case presentation

**Fig. 1:**
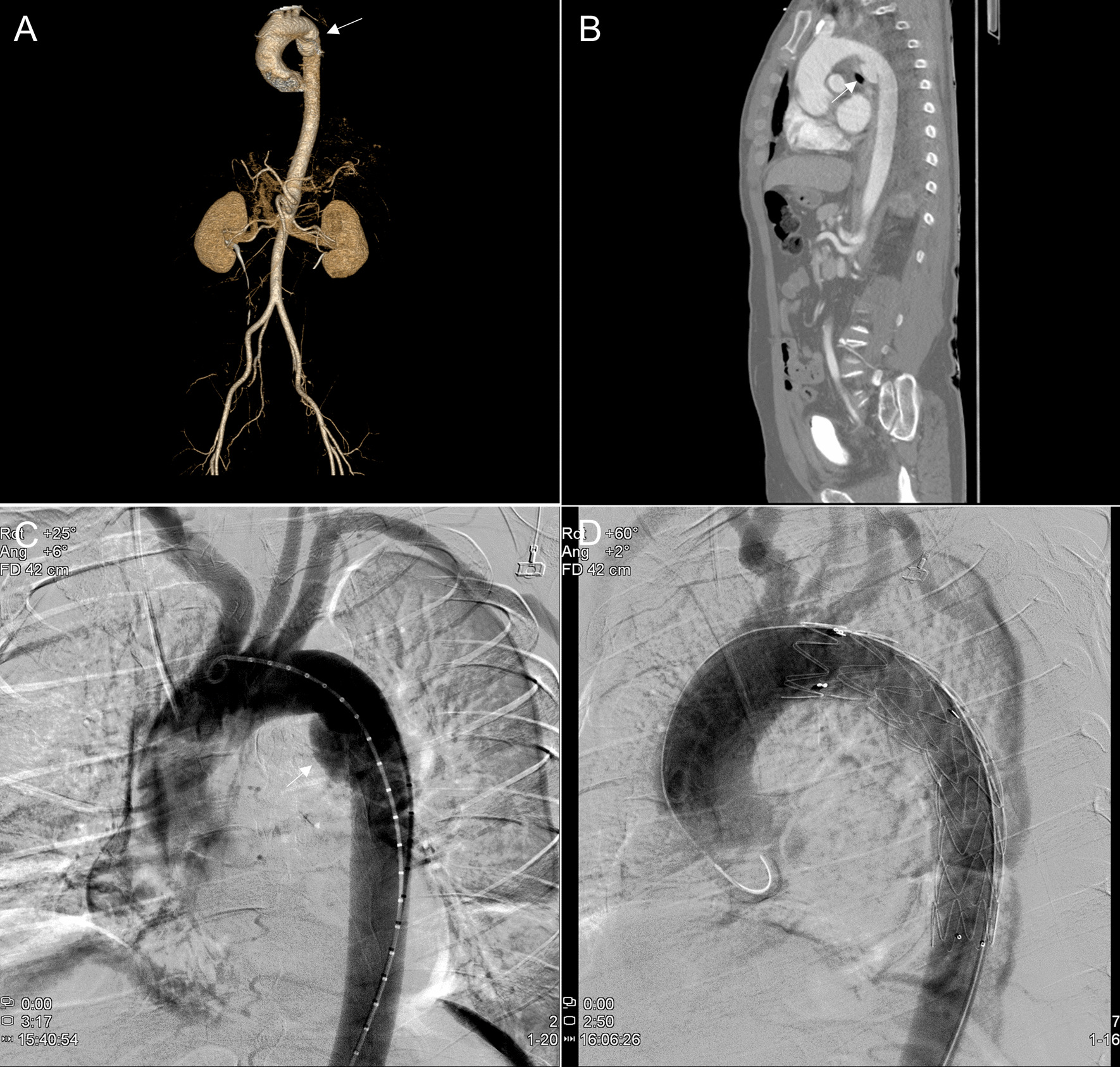
Computed tomography of aorta and aortogram of patient and aortogram performed after stent graft insertion. **A** 3D reconstruction image of patient’s aorta computed tomography. **B** Sagittal image of patient’s aorta computed tomography. **C** Aortogram of patient’s aorta. **D** Aortogram performed after stent graft insertion. White arrow indicates saccular dissection of descending thoracic aorta

**Fig. 2:**
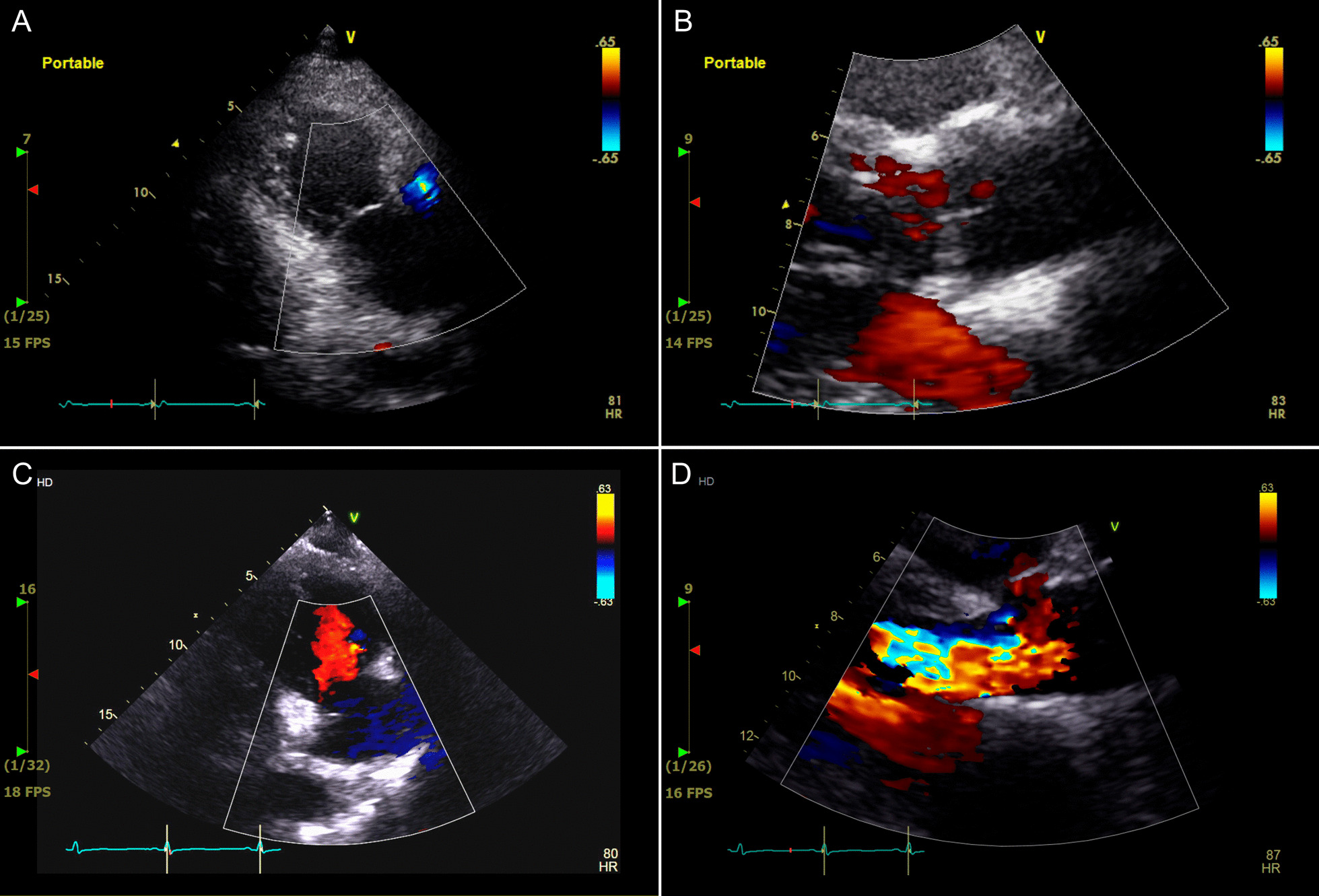
Echocardiograms performed at the time of aorta injury and 42 months after stent graft insertion. **A**–**B** An echocardiogram performed on the fourth day after TEVAR (EF 61%, LVEDD/LVESD 51/34mm, LA 40 mm, LV mass index 107.5 g/m2, Aortic annulus/Sinus valsalva/Sinotubular junction/Ascending aorta 22 mm/38mm/31mm/38mm, Trivial MR, Trivial TR). **C**–**D** An echocardiogram performed at the emergency room for sudden dyspnea 42 months after stent graft insertion (EF 63%, LVEDD/LVESD 90/46mm, LA 59 mm, LV mass index 169.7 g/m2, Aortic annulus/Sinus valsalva/Sinotubular junction/Ascending aorta 22 mm/35mm/27mm/ 40 mm, AR grade 4, MR grade 1, Trivial TR). (*EF* Ejection fraction, *LVEDD* Left ventricular end diastolic dimension, *LVESD* Left ventricular end systolic dimension, *LA* Left atrium, *LV* Left ventricle, *MR* Mitral valve regurgitation, *TR*  Tricuspid valve regurgitation,  *AR* Aortic valve regurgitation)

**Fig. 3:**
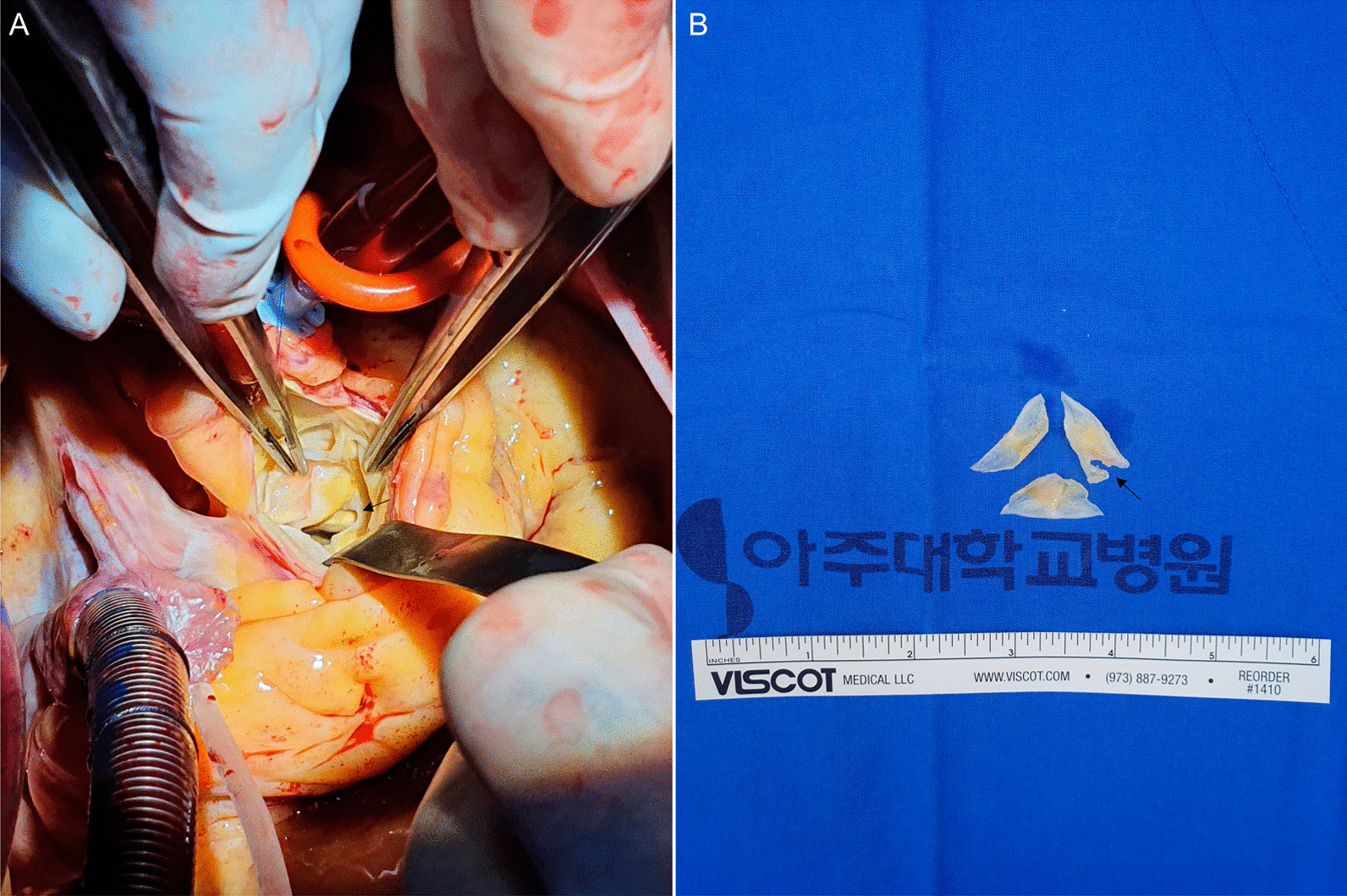
Aortic valve injury confirmed in the operating room and morphology of resected aortic valves. **A** Injured aortic valve confirmed after cardiopulmonary bypass before aortic valve replacement. Black arrow indicates injured aortic valve of right coronary cusp. **B** Morphology of resected aortic valves. Black arrow indicates injured aortic valve of right coronary cusp

The 58-year-old male patient visited a local hospital for traffic accident by bicycle and was transferred to this medical institution with isthmus portion saccular dissection of descending thoracic aorta (Fig. 1 A–C) and performed aortic stent graft insertion (Fig. 1D, Additional file : 1). Briefly, after ultrasound-guided femoral puncture, a Lunderquist extra-stiff wire (Cook Medical, Bloomington, IN, USA) was introduced. Then, a stent graft (Valiant Captivia; 32–28 mm, tapered; length: 1150 mm; Medtronic, Minneapolis, MN, USA) was inserted along the pre-inserted wire. On the 7th day after the procedure, F/U aorta CT showed no endo-leakage of the stent graft and was discharged without any special complications. During the outpatient follow-up, no unusual findings were observed at 3 months, 9 months, 15 months, and 27 months follow-up. Severe aortic regurgitation with flail aortic valve movement (right coronary cusp), which was not observed when TEVAR treatment (Fig. 2 A–B, Additional file :  2), was observed on an echocardiogram performed through the emergency room of the hospital due to sudden dyspnea 42 months after the stent graft operation (Fig. 2 C–D, Additional file :  3). Resected sharp margin of the right coronary cusp was observed in the aortic valve during surgery (Fig. 3 A–B). For this, aortic valve replacement (Edwards tissue aortic valve#23mm) was performed and the patient was discharged on the 8th day of POD without any special complications.

## Discussion and conclusions

TEVER was initially attempted in a situation where it could not be a surgical candidate, but as the procedure technique and materials have been developed, it is being considered as a therapeutic option in various situations [3]. However, it is also true that there are no prospective randomized trials for TEVAR trials. There are no long-term data compared with the open surgical approach yet, but it is a known fact that morbidity and mortality are superior in most cases, so the treatment range is highly likely to expand in the future.

Arterial dissection, iliac artery rupture, and arterial perforation are classified as complications as possible device delivery injuries during TEVAR implementation [5]. However, there is no literature review on catheter-induced aortic valve injury related to TEVAR. Recently, the bioprosthetic aortic acallop intentional laceration to prevenet coronary artery obstruction (BASILICA) procedure, a transcatheter laceration of aortic leaflet procedure, is being introduced to prevent coronary ostium obstruction that occurs after transcatheter aortic valve replacement [6]. This procedure is rather considered to be an important case showing that an aortic valve injury can occur at any time if care is not taken during the TEVAR procedure.

It is possible that the AR developed during the traffic accident. However, based on the following points, it is likely that the patient had delayed traumatic AR caused by TEVAR procedure. First, when multiple chest wall fractures occur, the traction force to the aortic isthmus is reduced; however, the aortic blood column rapidly decelerates, delivering a shock to the aortic valve and causing rupture [7, 8]. Our patient did not have multiple fractures, preventing heart displacement during trauma, leading to aortic stretching and isthmus injury, suggesting that AR was not caused by the traffic accident. Second, it has been known that traumatic AR found that rupture most commonly involves the non-coronary cusp, although it can involve the right and left coronary cusp because of coronary perfusion system [9]. Third, previous studies have reported that trauma damages the bottom of the valve near its nadir because this is its thinnest portion and it is easily torn when subjected to high pressure [10, 11]. Our patient had injury to a relatively thick part of the valve (near commissure portion).

Most of the catheter-induced aortic valve injury cases have been reported during coronary angiography and percutaneous coronary intervention (PCI), but traumatic aortic valve injury is known to be a very rare complication (0.0001%) in this field as well [12]. Most of these cases cause acute onset aortic regurgitation, requiring urgent surgical intervention. According to a literature review of coronary angiography and PCI-induced severe aortic regurgitation in 2021 [13], A total of 15 cases have been reported so far, but most of them were acute form of severe aortic regurgitation that occurred immediately after the procedure. As in this study, the cases accompanied by laceration of the aortic valve by the catheter were only a case that occurred 24 months later and a case that occurred 8 months later.

First, this case is meaningful in that it is the first to report catheter-induced aortic valve injury that occurred during the TEVER process, and secondly, the aortic that occurred after the longest period (42 months) among all cases including coronary angiography and PCI-induced severe aortic regurgitation. When performing a catheter-related procedure around the aortic valve, even if no particular problem is observed immediately after the procedure, a weak valve injury can cause acute aortic valve insufficiency even after 3 years, so special attention is always required.

## Supplementary Information

**Additional file 1**. Aortogram performed before and after stent graft insertion.

**Additional file 2**. Echocardiogram of parasternal long/short axis view of aortic valve at the time of aorta injury.

**Additional file 3**. Echocardiogram of parasternal long axis view of aortic valve after stent graft insertion.

## Data Availability

Data sharing not applicable to this article as no datasets were generated or analysed in this study.

## Abbreviations

AR: Aortic regurgitation
LV: Left ventricle
TEVAR: Thoracic endovascular aortic repair
BASILICA: Bioprosthetic aortic acallop intentional laceration to prevenet coronary artery obstruction
PCI: Percutaneous coronary intervention
